# Indices reflecting muscle contraction performance during exercise based on a combined electromyography and mechanomyography approach

**DOI:** 10.1038/s41598-021-00671-2

**Published:** 2021-10-27

**Authors:** Shinichi Fukuhara, Takaki Kawashima, Hisao Oka

**Affiliations:** 1 Department of Medical Engineering, Faculty of Health Science and Technology, Kawasaki University of Medical Welfare, Kurashiki, Okayama 701-0193 Japan; 2 Department of Physical Therapist, Kawasaki Junior College of Rehabilitation, Kurashiki, Okayama 701-0192 Japan; 3 Graduate School of Interdisciplinary Science and Engineering of Health Systems, Okayama University, Okayama, 700-0082 Japan

**Keywords:** Biomedical engineering, Health care

## Abstract

Electromyography (EMG) and mechanomyography (MMG) have been used to directly evaluate muscle function through the electromechanical aspect of muscle contraction. The purpose of this study was to establish new absolute indices to describe muscle contraction performance during dynamic exercise by combining EMG and displacement MMG (dMMG) measured simultaneously using our previously developed MMG/EMG hybrid transducer system. Study participants were eight healthy male non-athletes (controls) and eight male athletes. EMG and dMMG of the vastus medialis were measured for 30 s during four cycles of recumbent bicycle pedaling (30, 60, 90, and 120 W) and on passive joint movement. Total powers were calculated based on the time domain waveforms of both signals. Muscle contraction performance was verified with the slope of regression line (SRL) and the residual sum of squares (RSS) obtained from EMG and dMMG correlation. EMG and dMMG has increased with the work rate. Force and EMG were similar between groups, but dMMG showed a significant difference with load increase. Athletes had significantly higher SRL and significantly lower RSS than controls. The average value divided by SRL and RSS was higher in athletes than in controls. The indices presented by the combined approach of EMG and dMMG showed a clear contrast between the investigated groups and may be parameters that reflect muscle contraction performance during dynamic exercise.

## Introduction

A non-invasive and simple evaluation method is desirable for examiners, athletes, and patients in order to understand the improvement in athletic performance due to daily training and treatment. Muscle function in exercise, sports, and rehabilitation situation is often evaluated using simple quantitative measures such as maximum muscle strength via muscle strength dynamometer and manual muscle test^1^, number of enforcement time^2^. However, these methods are greatly affected by effort, motivation, and experience of the examinee and may result in qualitative evaluation variability^3,4^. Many studies have attempted evaluation of muscle function during dynamic exercise on a laboratory scale using electromyography (EMG) and mechanomyography (MMG), which can directly evaluate the target muscles. EMG quantifies the extent to which nerve drive causes activation of motor units during muscle contraction^5^. MMG measured using various transducers such as accelerometers on the skin surface directly measures muscle displacement and vibrations during muscle contraction, and can be representative of the final proof of the muscle activity^6^. In other words, these evaluations can act as an input/output relationship measures during muscle contraction. Furthermore, there is dissociation when using combined evaluation via EMG and MMG between muscular dystrophy patients and healthy subjects^7^. This indicates a decrease in the electro-mechanical coupling efficiency of muscle due to the disease. A combined evaluation using EMG and MMG may reflect the overall performance of an individual muscle itself.

We hypothesized that the evaluation of muscle function using a combined approach with EMG and MMG reflects muscle contraction performance during dynamic exercise, highlighting individual differences in athletic ability, even among healthy subjects. There have been many reports on simultaneous measurement of EMG and MMG in dynamic exercise^8–10^. In particular, muscle activity analysis related to pedaling exercises investigating effects on the quadriceps femoris has been conducted in numerous reports^11–14^. However, many of these reports are independent evaluations based on non-standardized methodology, such as processed amplitude or mean power frequency (MPF). It is necessary to evaluate from both electrical and mechanical activities for accurate evaluation of muscle. Independent estimation of time-varying muscle strength is required for a wide range of movements envisioned in sports and rehabilitation. Ultrasound is expected to be used for direct measurement of muscle strength in terms of mechanical output^15^, but it is not clinically suitable because the morphology of the muscle can easily change depending on the examiner's skill and the amount of pressure on the probe, and the relative position of the probe to the muscle can change due to dynamic movement. In this respect, MMG has the advantage of easy placement of miniaturized sensors and direct and pinpoint measurement of mechanical information associated with minute morphological changes of muscles during exercise. However, there are no reports that mention complex and hybrid evaluations using EMG and MMG during dynamic exercise. Until now, simultaneous measurement of EMG and MMG has not described the intrinsic contraction performance of muscles. By establishing an index to represent muscle contraction performance, this combined approach enables a direct and absolute evaluation of a particular muscle rather than using conventional relative estimations, such as inter-subject measures. Hybrid evaluation of muscle based on EMG and MMG provide new valuable information on muscle function during exercise.

MMG remained at the research level due to some inconsistency regarding physical quantities measured by various conventional transducers and unsuitability for dynamic measurement, and was considered to be less versatile than EMG^16^. Recently, our group developed an MMG/EMG hybrid transducer system that simultaneously measures EMG and MMG on a portable single device^17^. One previous study showed that vastus medialis (VM) function could be quantitatively evaluated from the electro-mechanical aspect of muscle contraction during pedaling and expressed a potential for exploring muscle contraction performance using EMG and MMG^18^. The purpose of this study was to provide a unique and new evaluation index that reflects muscle contraction performance through a combined approach using EMG and MMG measured during exercise. The combined evaluation of EMG and MMG to objectively quantify muscle performance during dynamic exercise is simple and desirable in sports and rehabilitation and has the potential to be useful exercise-based training and treatment strategies planning.

## Methods

### Subjects

Table 1 lists the basic characteristics of the 16 male subjects (age: 20–22 years) included. Eight active athletes and eight non-athletes (controls) without specific athletic history and daily exercise participated in this study. Subjects were clinically healthy without previous injuries or comorbidities on medical history. The athletes' sports consisted of four activities: middle-distance running, rowing, and triathlon. The athletes trained routinely and their competition history spanned 5–10 years. This study was conducted according to the principles of the Declaration of Helsinki with ethical approval from the Kawasaki University medical welfare ethics committee (approval number 19-013). All subjects received sufficient explanation about the experiment and participated after obtaining informed consent.

**Table 1:** Basic anthropometric characteristics of control and athlete subjects.

	Controls	Athletes	p-value
Age (years)	21.3 ± 0.9	20.3 ± 0.5	0.013
Body height (cm)	166.6 ± 5.8	172.0 ± 5.9	0.076
Body weight (kg)	56.7 ± 8.9	63.6 ± 7.8	0.085
Body surface area (m^2^)	1.63 ± 0.11	1.76 ± 0.13	0.332
Body mass index (kg/m^2^)	20.4 ± 2.0	21.4 ± 1.9	0.058

Data are means ± SD.

### MMG/EMG hybrid transducer

**Figure 1:**
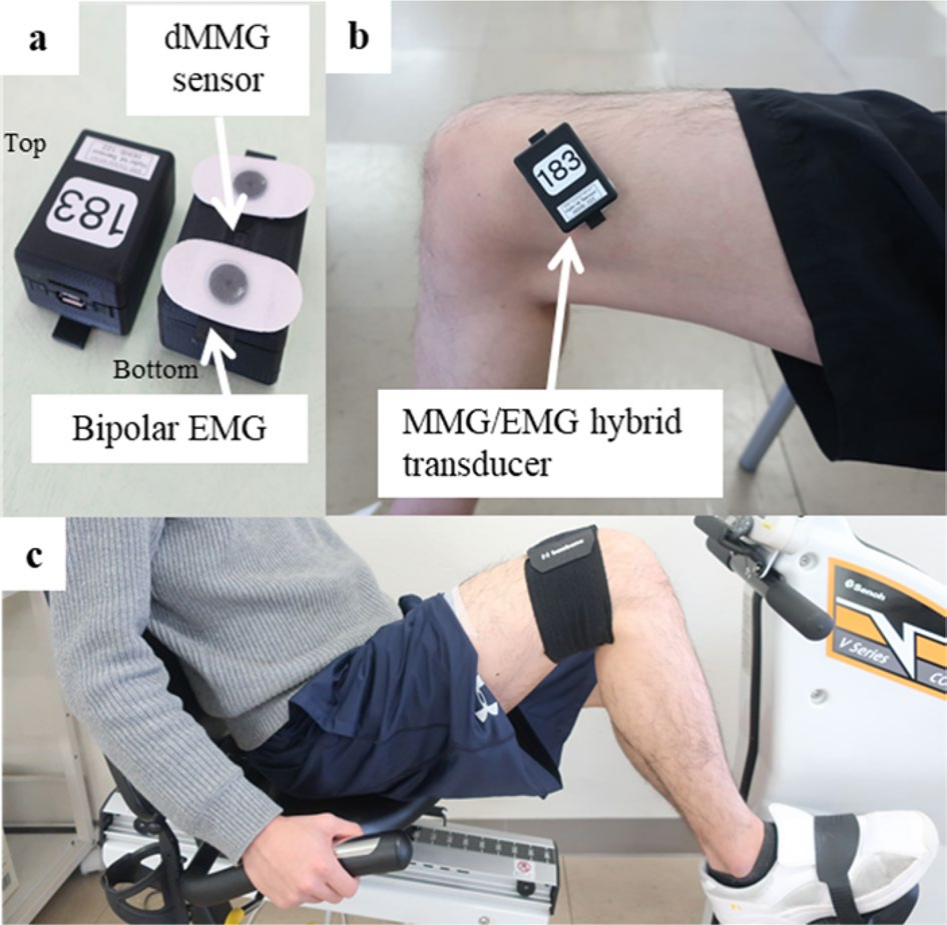
Example of measuring EMG and dMMG. (**a**) MMG/EMG hybrid transducer. (**b**) Attachment the MMG/EMG hybrid transducer to the skin surface of the vastus medialis. (**c**) Subject sits on the recumbent bicycle with both toes fixed to the pedals and both hands gripping the handles.

Figure 1a shows the MMG/EMG hybrid transducer (HOHS-122, ERD Co. Okayama, Japan) we developed. This transducer measures 47 × 34 × 24 mm and weighs 34 g. An MMG sensor is located in the center of the bottom of transducer and EMG disposable electrodes are attached to both ends. The photo reflector using the MMG sensor was designed to be 3 mm above the skin surface. Skin variation was recorded as the displacement MMG (dMMG). The dMMG measured using this system was calculated in advance based on calibrated distance-voltage characteristics in the 12-bit A/D conversion. The transducer and PC communicated via Bluetooth and data were recorded on a built-in SD card at any recording time and sampling frequency. EMG and dMMG measured by this transducer had stable signals during pedaling while not being affected by motion artifact. Although dMMG captures simple changes in muscle morphology during exercise when strength is exerted, it is possible to extract only net muscle strength components by normalizing data using dMMG for passive (involuntary) joint movement. The dMMG directly detects recruitment of muscle fibers by the physical quantity of length (mm) during exercise; that is, it represents changes in the physiological muscle cross-sectional area due to increased muscle contraction. We have proved that the work rate, EMG, and dMMG are in a linear relationship for a certain load using the developed transducer^18^. There is a recent study that uses this transformer to objectively quantify the patellar tendon reflex and use it to diagnose neurological diseases^19^.

### Experimental set-up

Prior to the pedaling, subjects attempted maximum muscle contraction with their right lower limbs fixed at 90°. Subjects were fixed to the backrest and performed knee extension exercises. Muscle strength was manually measured using a hand-held dynamometer (mTas F-1, ANIMA Inc., Tokyo, Japan) placed on the fore ankle. The sensor pad was placed on the fore ankle. Then, subject skin was prepared by dedicate gel application before attachment of the MMG/EMG hybrid transducer to reduce contact resistance. The transducer was attached on top of an affixed white marker to the center of the muscle of the right VM (Fig. 1b). Subjects were then seated on a recumbent bicycle (V67i, SENOH Corp., Chiba, Japan) and pedaled in a seated position after being secured with a dedicated belt to keep the transducer attached. The knee joint angle formed when the pedal was at the top dead center was set to 100°–110° along the coronal plane using a goniometer by sliding the seat of the recumbent bicycle. Their backs were firmly in close contact with the backrest, toes were fixed with a belt, and both hands gripped the hand grips (Fig. 1c). To eliminate the effects of antagonist muscles on pedaling, subjects were instructed to focus on depressing on pedal. The cadence was set to 30 rpm for both feet (15 rpm per side). Pedaling work rates were set at 30, 60, 90, and 120 W. Two experimenters manually rotated the pedals when in the subject’s involuntary muscle contraction (passive pedaling) testing phase. Subjects kept their cadence constant with the support of a metronome and their rotation speed was displayed on the recumbent bicycle. Pedaling was performed three times at each work rate per person. Sufficient rest time was provided between experiments. Data were sampled at 1 kHz for 30 s.

### Data analysis

Data were analyzed by adapting our previously reported method without conventional analysis (e.g., average rectified value [ARV] and root mean square [RMS])^20^. The EMG and dMMG data were processed in the time domain. All data were squared and were integrated every 2 s. As a result, 15 data sets were created, with corresponding averages defined as EMG_TD_ and dMMG_TD_. As in our previous studies, EMG_TD_ values were normalized from the maximum value for each subject (n-EMG_TD_), and dMMG_TD_ values were normalized from dMMG_TD_ data from passive pedaling (n-dMMG_TD_)^18^. The above series of analyses calculated the total power (energy) of EMG and dMMG during pedaling for 30 s. All final results comprised the average of pedaling for three cycles. Furthermore, the slope of the regression line (SRL) and the residual sum of squares (RSS) were calculated from the relationship between n-EMG_TD_ and n-dMMG_TD_ for each individual. Finally the SRL was divided by the RSS. This value was defined as dynamic muscle performance index (DMPI), which determines muscle contraction performance during exercise.

### Statistical analysis

Data were expressed as mean ± SD. Statistical analysis of the data was performed using SPSS Statistics 24.0 (IBM, Armonk, NY, USA). The normality of the data was evaluated using the Shapiro–wilk test. Differences between the two groups (athletes versus controls) and within-subjects across the work rate were assessed by two-way factorial ANOVA; if a significant difference was observed, intergroup comparisons were performed with post-hoc Sidak test (alpha value of 0.05). In addition, a student’s t test was performed to compare force, SRL and RSS for each load level, and DMPI between the two investigated groups (alpha value of 0.05, two sided). The correlations between n-EMG_TD_ and n-dMMG_TD_ were investigated using the Pearson correlation test (alpha value of 0.05, two sided).

## Results

### Force, EMG_TD_, and dMMG_TD_

The force measured by the manual strength testing was not significantly different between athletes (450.7 ± 114.1 N) and the controls (384.3 ± 120.0 N).

**Figure 2:**
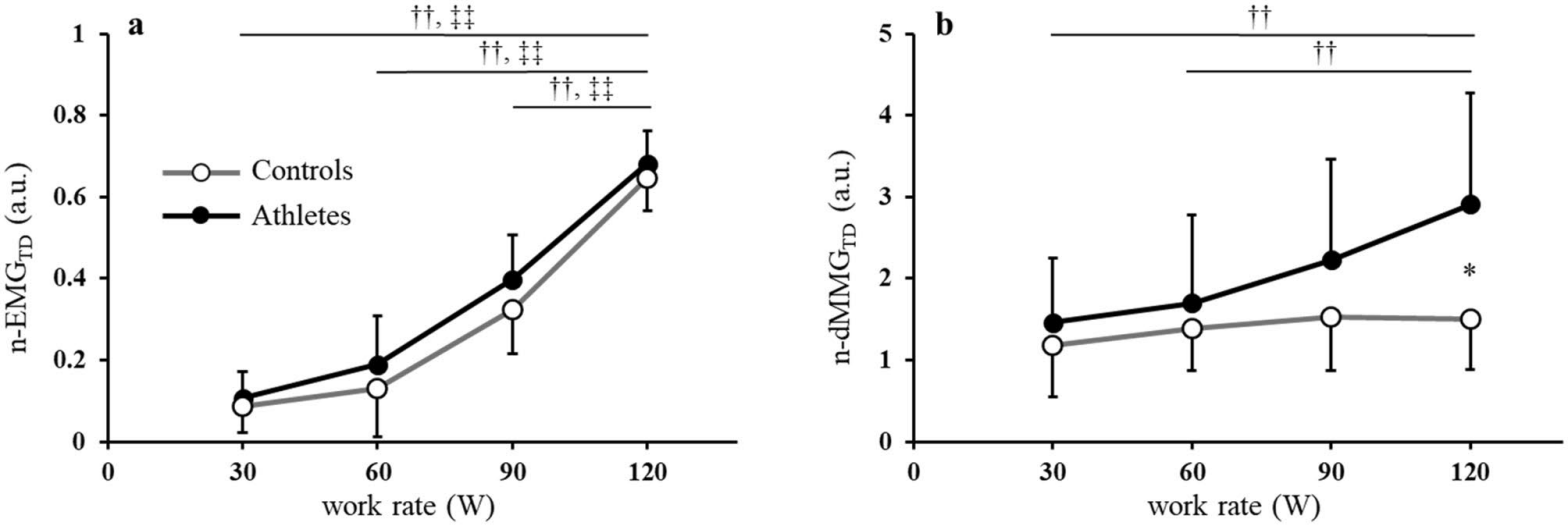
Relationship between work rate and (**a**) n-EMG_TD_, (**b**) n-dMMG_TD_, in controls (open circles) and athletes (closed circles). All quantitative data are expressed as means ± SD; n = 8, 8 for control and athlete groups, respectively. *p < 0.05 vs. controls. ^††^p < 0.01: within MMG subjects, ^‡‡^p < 0.01: within EMG subjects.

In Fig. 2a, the mean values of n-EMG_TD_ are plotted as a function of each work rate for athletes and controls. In both groups, n-EMG_TD_ gradually increased in a similarly shaped curve with work rate. No differences were found between athletes and controls at any work rate.

The mean value of n-dMMG_TD_ as a function of each work rate for athletes and controls are shown in Fig. 2b. In controls, n-dMMG_TD_ was slightly increased or displayed almost no change across all work rates. In contrast, n-dMMG_TD_ of athletes increased linearly from 30 to 120 W. Moreover, at 120 W, n-dMMG_TD_ was considerable higher in athletes than in controls.

### Relationship between EMG_TD_ and dMMG_TD_

SRL, RSS (to evaluate the difference between the linear regression model and the data), and the correlation coefficient for all athlete and control subjects are presented in Table 2. All athlete subjects had a strong positive correlation from 0.522 to 0.867. The controls had a weaker correlation coefficient than the athletes. Some subjects had no correlation. The SRL and RSS values were higher in athletes than in controls. On the other hand, the RSS was lower for athletes. These results indicate that high mechanical activity against electrical activity is stably exerted from 30 to 120 W in athletes.

**Table 2:** Data of correlation coefficient, SRL, RSS for each subject, in controls and athletes, and average values of SRL and RSS for both groups.

Controls				Athletes			
Subject	SRL	RSS	Correlation coefficients	Subject	SRL	RSS	Correlation coefficients
1	0.479	2.416	0.508**	9	3.000	1.878	0.694**
2	0.849	2.577	0.478**	10	2.730	1.142	0.867**
3	0.136	3.693	0.135	11	2.641	1.467	0.768**
4	0.025	3.863	0.328*	12	2.266	1.641	0.815**
5	0.959	1.570	0.750**	13	2.915	1.066	0.867**
6	-0.443	4.976	− 0.229	14	1.058	1.334	0.703**
7	0.243	3.461	0.456**	15	1.434	1.768	0.732**
8	0.085	2.665	0.06	16	1.041	2.776	0.522**
Means ± SD	0.292 ± 0.458	3.152 ± 1.058			2.136 ± 0.831^††^	1.634 ± 0.543^††^	

*p < 0.05, **p < 0.01: statistical significance for correlation coefficient. ^††^p < 0.01: vs. controls.

**Figure 3:**
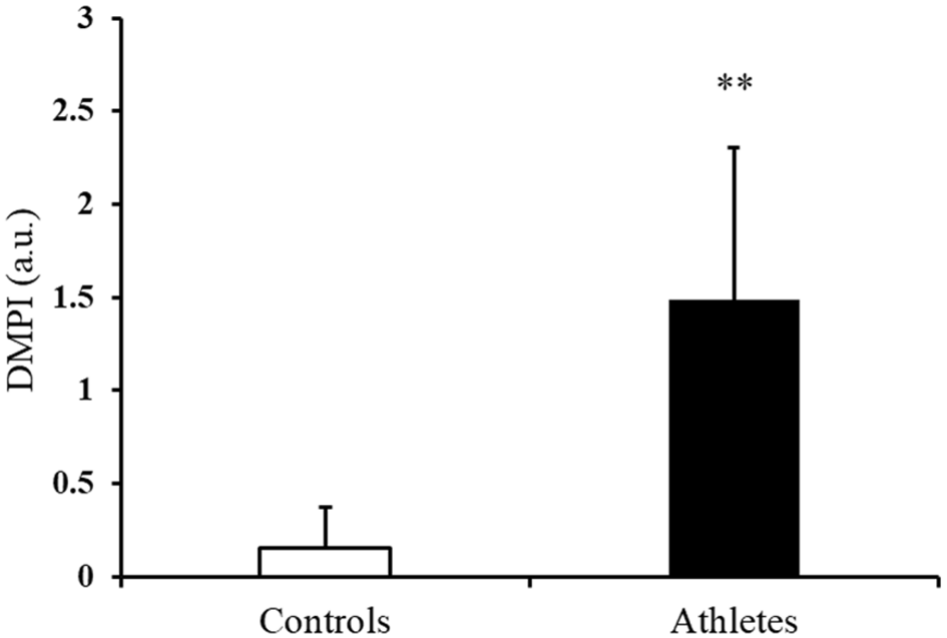
Average of SRL divided by RSS, i.e. DMPI in controls (white column) and athletes (black column). Data are expressed as means ± SD. Statistical significance; **p < 0.01.

The average value of DMPI for athletes and controls is shown in Fig. 3. The DMPI was 113% higher in athletes than in controls and showed a clear contrast.

## Discussion

In general, muscle performance in exercise is expected to be higher in athletes who engage in sports on a daily basis than in non-exercisers. Several reports have evaluated the muscle function of sports athletes from various aspects. Muscle function of athletes is characterized by muscle mass^21,22^, stiffness^23^, and muscle strength^24^. Training improves the central nervous system and causes muscle hypertrophy due to an increase in the number of myofibrils. In addition, athletes are considered to have clear differences in the size and recruitment mechanism of their muscle motor units during exercise.

Under conditions that exclude constraints such as fatigue and nonlinear force exertion caused by muscle viscoelasticity, EMG in healthy people is an indicator that reflects the muscle strength^25^. All subjects maintained pedaling under constant load and cadence conditions in this experiment. Therefore, the muscle strength required to maintain pedaling must be constant regardless of subject. Assuming that the contribution of VMs to pedaling is equal in all subjects, it is a reasonable result that there was no difference between the two groups regarding EMG_TD_, which is the input signal for muscle contraction. Indeed, EMG during dynamic exercise estimates muscle strength, but it is difficult to normalize (e.g., maximum voluntary contraction [MVC]) under certain dynamic exercise conditions. It is assumed that EMG is inadequate for estimating an individual's muscle contraction performance during pedaling. The EMG signal obtained in the present study was not sensitive to determining the muscle function. On the other hand, MMG represents the final output of the muscle contraction to the input signal in that the higher the MMG, the more motor units are recruited, and it captures the physical behavior of muscle fibers during muscle contraction^26^. MMG reflects the state of the mechanical activity of muscles. Akataki et al. reported that the MMG of the quadriceps femoris measured by an accelerometer in isometric contraction correlates with muscle strength at 10–80% MVC and shows a decreasing tendency thereafter^27^. In this study, n-dMMG_TD_ of the controls increased slightly with pedaling work rate and became constant from 90 W upwards. This result suggests that the motor unit completed mobilization in the range of low load compared to the athlete, and the pedaling was maintained at a constant level due to an increase in synapse firing rate in the subsequent load. On the other hand, n-dMMG_TD_ continued to increase proportionally with pedaling work rate in athletes. This result suggests that n-dMMG_TD_ illustrates the recruitment of motor units still in progress even at the maximum load and that athletes have a margin of reserve muscle strength for the exercise compared to controls. In particular, the triathlon athletes in this study may have a better muscle function for pedaling than other athletes because of their regular specialized pedaling training. It is significant that n-dMMG_TD_ showed the superiority of athletes. Previous studies have examined the amplitude of MMG based on sex differences^28^ and age^29^ in isometric contraction. MMG of men and youths, who are supposed to have higher athletic ability, were higher in a range of relatively large loads. However, muscle contraction performance using EMG and MMG measured during exercise can be understood only by considering factors such as inter-subject factors, inter-muscle factors, and time; that is, there is no absolute index to express muscle contraction performance.

Until now, competitive performance during pedaling exercise has been estimated by indirect indicators measured by devices via joint movement such as maximum power in full-power pedaling^30^, cadence at maximum power^31^, and pedaling force^32^, with no absolute index showing muscle-specific characteristics. EMG and MMG can be mentioned as alternatives to the above, but both signals are evaluated independently using diverse evaluation items and must be evaluated relative to each other as mentioned earlier. Additionally, the relationship between force and EMG amplitude has been widely used as an indicator of electro-mechanical activity during muscle contraction in traditional research^33,34^; however, this measured force is ultimately an indirect indicator through joint movement. Several researchers have demonstrated an EMG-MMG combined approach during isometric contraction in patients with muscular dystrophy or myogenic disease^7,35^. All studies reported that the EMG-MMG ratio was lower in patients with disease than in healthy subjects, suggesting that electromechanical coupling efficiency of muscle in patients with disease is reduced. Conversely, it is speculated that muscles can contract more efficiently as the EMG-MMG ratio increases. It is assumed that muscles suitable for exercise can produce a higher MMG (output) relative to the EMG (input) value during the muscle contraction process. Naturally, muscles unsuitable for exercise will display the opposite. For the above reason, we hypothesized that the EMG-MMG ratio determines the superiority or inferiority of the individual muscle contraction performance in exercise. In fact, the SRL of the athlete group was significantly higher than that of the controls (Table 2). The slopes obtained from this dynamic exercise were comparable to the EMG-MMG ratio of previous EMG-MMG combined studies and, as a result, indicate that a high electro-mechanical coupling efficiency, a characteristic that allows it to produce a large output (dMMG) for an input (EMG). Sport athletes are expected to have different composition ratios of muscle fibers, and recruitment patterns of motor units compared to ordinary people because of their characteristics of competition, training, and other innate qualities^36,37^. Since SRL represents the ratio of output/input (n-dMMG_TD_/n-EMG_TD_) in muscle contraction, it may represents the efficiency of muscle contraction. An efficient muscle means that it produces more work with less input energy. Several studies reported that people with a high proportion of type I muscle fibers have higher exercise efficiency than those with a high proportion of type II muscle fibers^38–40^. The activities performed by the athletes in this study required relatively good endurance, and the SRL of the athlete group may have been higher than that of the control group. Additionally, Esposito et al. reported a clear difference in EMG and MMG amplitudes between elite rock climbers and controls^41^. It seemed that the SRL calculated from EMG_TD_ and dMMG_TD_ in dynamic exercise represents a part of the performance of muscle contraction with respect to exercise.

On the other hand, we focused on the extent to which the analyzed EMG_TD_ and dMMG_TD_ fit a regression line. The RSS is an index that evaluates the difference between measured (analyzed) data and an estimated model. A lower RSS indicates that the model fits snugly against the data. The RSS of athletes was significantly lower than that of controls (Table 1). This means that athletes had a stable dMMG_TD_-to-EMG_TD_ signal to maintain dynamic repetitive and cyclic movement. The amount of change in the muscle expansion and contraction (physiological cross-sectional area) captured by the raw dMMG seems to consistently increase and decrease during pedaling with knee flexion and extension. RSS may reflect the physiological properties of muscle viscoelasticity (due to changes in muscle morphology) apart from the output characteristic. Force steadiness in isometric muscle contraction has been investigated as an indicator of the accuracy of human exertion by a number of previous researchers^42,43^. The exerted force during voluntary muscle contraction fluctuates around the required force magnitude^44^. Elderly individuals have less force steadiness than youths^45,46^. Thus, stability of muscle contraction in response to constant exercise tasks may be one of the most important factors associated with muscle contraction performance. At constant velocity and load tasks in this study, the muscles of highly athletic people not only had high EMG-MMG ratio characteristics, but also provided a strong stability (low RSS) to sustain exercise conditions. Therefore, it is assumed that high muscle contraction performance requires higher SRL and lower RSS. It seems that these indicators express the ability to rapidly and stably achieve a constant cadence against the imposed load. Therefore, we proposed the DMPI during dynamic exercise in combination with SRL and RSS, which considers both characteristics. There was a clear difference in the DMPI of athletes versus that of controls (Fig. 3). These indices support the hypothesis of this study and may reflect the excellent muscle control function of athletes.

Finally, we demonstrated the relationship between indices of SRL, RSS, and DMPI and muscle ability using the MMG/EMG hybrid transducer system in this study. To the best of our knowledge, this study is the first to evaluate the muscle function using a new index that combines EMG and MMG during dynamic exercise. From a practical perspective, SRL and RSS have the advantage of not requiring complicated calculations. These indices may be new absolute indices that reflect muscle contraction performance in dynamic exercise.

The application of the indices presented, SRL, RSS, and DMPI, is currently confined to cyclic exercise with few disturbance elements and restricted joint movements. The analysis of muscle contraction performance during walking or running in daily movements and special movements in competitive sports remains a matter to be discussed further. Slight differences in individual exercise style, habits, and intensities may affect the indices of muscle contraction performance. Concerning this point, we believe that cyclic exercise that can exclude those effects is immensely effective in simply estimating muscle-specific performance. However, these indicators are possibility of vary greatly depending on the exercise load imposed on the subject. It may be necessary to develop an appropriate protocol for estimating muscle contraction performance.

**Figure 4:**
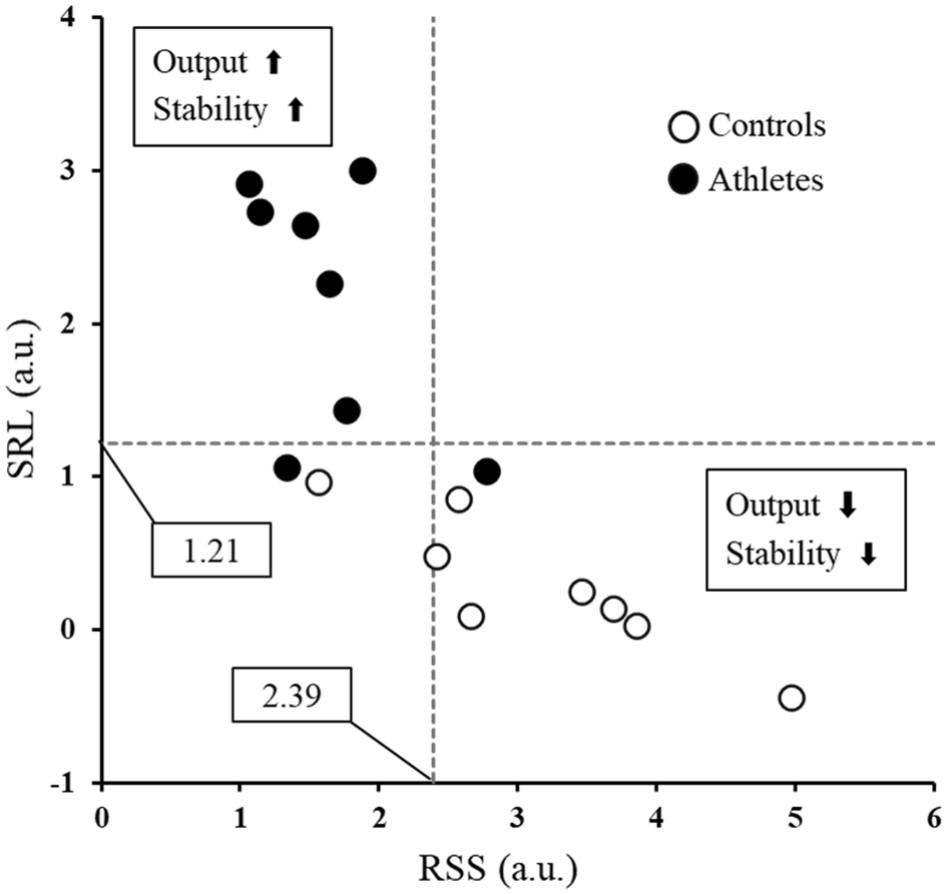
A classification indication muscle contraction performance, in controls (open circles) and athletes (closed circles). The numbers in the squares and dotted lines are the average of all subjects on each axis. The upper left category has superior both characteristics of responsiveness and stability, which are inferior in the lower right.

The focus of the present study was that several indices obtained using a combined evaluation of EMG and dMMG can evaluate the performance of individual muscles. We expect these indices to represent the potential ability of muscles during dynamic exercise. In rehabilitation and nursing care situations, a motor function evaluation method during practical movement is required rather than static evaluation^47,48^. Figure 4 shows the muscle contraction performance of all subjects in this study classified using SRL and RSS, with average values shown in the figure. Higher SRL and lower RSS are plotted in the upper left section of the figure, representing higher performance muscles. It seems possible to depict the superiority or inferiority of an individual's athletic ability according to muscle contraction performance in four zones. This classification extracts detailed characteristics of the target muscle and may contribute to the evaluation of variables, such as athlete competitiveness and rehabilitation effectiveness; however, we must look more carefully into the interpretation of exercise physiology of each classification. In order to establish and bolster the indices proposed in this study in practical application, a further direction of this study would be to recruit more subjects of diverse backgrounds, such as sex, age, height, weight, and exercise history, to create more variable data distribution.

In conclusion, this study demonstrated the effectiveness of evaluating dynamic muscle contraction performance using various indices, which were obtained using a combined approach with EMG and dMMG, obtained via the MMG/EMG hybrid transducer system, during recumbent bicycle pedaling. The indices showed a clear contrast between the controls and athletes in a range of low-to-high exercise loads and could be parameters that reflect potential muscle-specific performance during dynamic exercise.

## Data Availability

The data that support the findings of this study are available from the corresponding author (S.F.).

## References

[1] Wadsworth CT, Krishnan R, Sear M, Harrold J, Nielsen DH (1987). Intrarater reliability of manual muscle testing and hand-held dynametric muscle testing. Phys. Ther..

[2] Thaweewannakij T (2013). Reference values of physical performance in Thai elderly people who are functioning well and dwelling in the community. Phys. Ther..

[3] Schwartz S, Cohen ME, Herbison GJ, Shah A (1992). Relationship between two measures of upper extremity strength: manual muscle test compared to hand-held myometry. Arch. Phys. Med. Rehabil..

[4] Bohannon RW (2005). Manual muscle testing: Does it meet the standards of an adequate screening test?. Clin. Rehabil..

[5] De Luca, C. Electromyography. *Encyclopedia of medical devices and instrumentation* (2006).

[6] Orizio C (1993). Muscle sound: Bases for the introduction of a mechanomyographic signal in muscle studies. Crit. Rev. Biomed. Eng..

[7] Barry DT, Gordon KE, Hinton GG (1990). Acoustic and surface EMG diagnosis of pediatric muscle disease. Muscle Nerve.

[8] Madeleine P, Bajaj P, Sogaard K, Arendt-Nielsen L (2001). Mechanomyography and electromyography force relationships during concentric, isometric and eccentric contractions. J. Electromyogr. Kinesiol..

[9] Beck TW (2005). Mechanomyographic amplitude and frequency responses during dynamic muscle actions: A comprehensive review. Biomed. Eng. Online.

[10] Anders JPV (2019). Inter- and intra-individual differences in EMG and MMG during maximal, bilateral dynamic leg extensions. Sports.

[11] Shinohara M, Kouzaki M, Yoshihisa T, Fukunaga T (1997). Mechanomyography of the human quadriceps muscle during incremental cycle ergometry. Eur. J. Appl. Physiol. Occup. Physiol..

[12] Housh TJ (2000). Mechanomyographic and electromyographic responses during submaximal cycle ergometry. Eur. J. Appl. Physiol..

[13] Perry SR (2001). Mean power frequency and amplitude of the mechanomyographic and electromyographic signals during incremental cycle ergometry. J. Electromyogr. Kinesiol..

[14] Bergstrom HC (2013). Mechanomyographic and metabolic responses during continuous cycle ergometry at critical power from the 3-min all-out test. J. Electromyogr. Kinesiol..

[15] Dick TJM, Biewener AA, Wakeling JM (2017). Comparison of human gastrocnemius forces predicted by Hill-type muscle models and estimated from ultrasound images. J. Exp. Biol..

[16] Watakabe M, Mita K, Akataki K, Ito K (2003). Reliability of the mechanomyogram detected with an accelerometer during voluntary contractions. Med. Biol. Eng. Comput..

[17] Oka H, Konishi Y, Kitawaki T (2014). Simultaneous measurement of displacement-MMG/EMG during exercise. SICE J. Control Meas. Syst. Integr..

[18] Fukuhara S, Watanabe S, Oka H (2018). Novel mechanomyogram/electromyogram hybrid transducer measurements reflect muscle strength during dynamic exercise: Pedaling of recumbent bicycle. Adv. Biomed. Eng..

[19] Tsuji H (2021). Quantification of patellar tendon reflex using portable mechanomyography and electromyography devices. Sci. Rep..

[20] Fukuhara S, Oka H (2019). A simplified analysis of real-time monitoring of muscle contraction during dynamic exercise using an MMG/EMG hybrid transducer system. Adv. Biomed. Eng..

[21] McCall GE, Byrnes WC, Dickinson A, Pattany PM, Fleck SJ (1996). Muscle fiber hypertrophy, hyperplasia, and capillary density in college men after resistance training. J. Appl. Physiol..

[22] Folland JP, Williams AG (2007). The adaptations to strength training: Morphological and neurological contributions to increased strength. Sports Med..

[23] Miyamoto N, Hirata K, Inoue K, Hashimoto T (2019). Muscle stiffness of the vastus lateralis in sprinters and long-distance runners. Med. Sci. Sports Exerc..

[24] Lesmes G, Costill D, Coyle E, Fink W (1978). Muscle strength and power changes during maximal isometric training. Med. Sci. Sports.

[25] De Luca CJ (1997). The use of surface electromyography in biomechanics. J. Appl. Biomech..

[26] Orizio C, Perini R, Diemont B, Maranzana Figini M, Veicsteinas A (1990). Spectral analysis of muscular sound during isometric contraction of biceps brachii. J. Appl. Physiol..

[27] Akataki K, Mita K, Itoh Y (1999). Relationship between mechanomyogram and force during voluntary contractions reinvestigated using spectral decomposition. Eur. J. Appl. Physiol. Occup. Physiol..

[28] Nonaka H, Mita K, Akataki K, Watakabe M, Itoh Y (2006). Sex differences in mechanomyographic responses to voluntary isometric contractions. Med. Sci. Sports Exerc..

[29] Tian S-L, Liu Y, Li L, Fu W-J, Peng C-H (2010). Mechanomyography is more sensitive than EMG in detecting age-related sarcopenia. J. Biomech..

[30] Davies CTM, Sandstrom ER (1989). Maximal mechanical power output and capacity of cyclists and young adults. Eur. J. Appl. Physiol. Occup. Physiol..

[31] Dorel S (2005). Torque and power-velocity relationships in cycling: Relevance to track sprint performance in world-class cyclists. Int. J. Sports Med..

[32] Dorel S, Drouet J-M, Couturier A, Champoux Y, Hug F (2009). Changes of pedaling technique and muscle coordination during an exhaustive exercise. Med. Sci. Sports Exerc..

[33] Alkner BA, Tesch PA, Berg HE (2000). Quadriceps EMG/force relationship in knee extension and leg press. Med. Sci. Sports Exerc..

[34] Doheny EP, Lowery MM, Fitzpatrick DP, O'Malley MJ (2008). Effect of elbow joint angle on force-EMG relationships in human elbow flexor and extensor muscles. J. Electromyogr. Kinesiol..

[35] Orizio C (1997). Muscle surface mechanical and electrical activities in myotonic dystrophy. Electromyogr. Clin. Neurophysiol..

[36] Costill DL, Fink WJ, Pollock ML (1976). Muscle fiber composition and enzyme activities of elite distance runners. Med. Sci. Sports.

[37] Andersen P, Henriksson J (1977). Capillary supply of the quadriceps femoris muscle of man: Adaptive response to exercise. J. Physiol..

[38] Horowitz J, Sidossis L, Coyle E (1994). High efficiency of type I muscle fibers improves. Int. J. Sports Med..

[39] Hunter GR, Newcomer BR, Larson-Meyer DE, Bamman MM, Weinsier RL (2001). Muscle metabolic economy is inversely related to exercise intensity and type II myofiber distribution. Muscle Nerve.

[40] Scheuermann BW, Tripse McConnell JH, Barstow TJ (2002). EMG and oxygen uptake responses during slow and fast ramp exercise in humans. Exp. Physiol..

[41] Esposito F (2009). Electrical and mechanical response of finger flexor muscles during voluntary isometric contractions in elite rock-climbers. Eur. J. Appl. Physiol..

[42] Enoka RM, Burnett RA, Graves AE, Kornatz KW, Laidlaw DH (1999). Task- and age-dependent variations in steadiness. Prog. Brain Res..

[43] Sorensen TJ (2011). The association between submaximal quadriceps force steadiness and the knee adduction moment during walking in patients with knee osteoarthritis. J. Orthop. Sports Phys. Ther..

[44] Enoka RM (2003). Mechanisms that contribute to differences in motor performance between young and old adults. J. Electromyogr. Kinesiol..

[45] Laidlaw DH, Bilodeau M, Enoka RM (2000). Steadiness is reduced and motor unit discharge is more variable in old adults. Muscle Nerve.

[46] Tracy BL, Enoka RM (2002). Older adults are less steady during submaximal isometric contractions with the knee extensor muscles. J. Appl. Physiol..

[47] Mahoney FI, Barthel DW (1965). Functional evaluation: the Barthel Index: A simple index of independence useful in scoring improvement in the rehabilitation of the chronically ill. Md. State Med. J..

[48] Heinemann AW, Michael Linacre J, Wright BD, Hamilton BB, Granger C (1994). Measurement characteristics of the functional independence measure. Top. Stroke Rehabil..

